# Correlation between skin test results and historical manifestations in patients with suspected lidocaine hypersensitivity

**DOI:** 10.5339/qmj.2022.fqac.16

**Published:** 2022-03-28

**Authors:** Sally Khalil, Salma Taha, Maryam Al-Nesf

**Affiliations:** ^1^Allergy Immunology division, Department of Medicine, Hamad Medical Corporation, Doha, Qatar E-mail: SKhalil3@hamad.qa

**Keywords:** hypersensitivity, lidocaine, skin prick test

## Abstract

Background: Adverse reactions to local anesthetics (LA) are relatively common; however, true IgE-mediated allergy is extremely rare, estimated to occur in less than 1%. Investigating patients with suspected allergy to LA should begin with a detailed history to exclude other more common operation theater related culprit medications, followed by skin testing. The subcutaneous challenge is considered the gold standard for confirming true IgE-mediated allergy to LA. In this study, we have described the skin prick test results of patients with suspected lidocaine allergy who had historical reaction symptoms typical to IgE-mediated allergic reactions.

Methods: The data were retrieved from the allergy procedure log registry for patients who were referred to the allergy clinic with a suspected allergic reaction to lidocaine at the Hamad Medical Corporation between 2016 and 2020. These patients’ symptoms of historical reactions to lidocaine were compared to their skin test results.

Result: A total of 7 patients were identified. The skin test result for lidocaine was positive in only 1 patient; his historical reaction was anaphylaxis (urticaria/angioedema and shortness of breath). The remaining 6 patients had a negative result for skin and challenge tests. Of these 6 patients with negative results, 4 had only urticaria/angioedema as historical reactions; 1 had systematic manifestation (tachycardia) along with urticaria/angioedema, and 1 experienced systemic symptoms (shortness of breath, chest pain, and palpitation) with no skin or mucous membrane involvement (Table 1).

Conclusion: Negative skin test and subcutaneous challenge with a history of generalized cutaneous symptoms and/or systemic symptoms during the reaction to LA can be attributed to many causes, such as an IgE-mediated reaction against a component other than lidocaine (e.g., latex), medication side effects (adrenaline in combined preparations), and/or symptoms of primary disease (chronic spontaneous urticaria/angioedema).

## Figures and Tables

**Table 1:** Detailed characteristics of historical reactions to LA; lidocaine

Patient	Age (years)	Gender	Allergic disease	Historical reaction	Time to onset	Skin test result	Drug challenge result

1	14	Female	None	Red itchy Jaw swelling	< 5 mins	–	–

2	27	Female	Chronic angioedema	Urticaria/ Angioedema	>1 hour	–	–

3	29	Female	CSU*, asthma	Throat tightness	>1 hour	–	–

4	31	Female	None	Anaphylaxis	>1 hour	–	–

5	32	Female	Other drugs allergy	Urticarial rash	Unknown	–	–

6	40	Female	None	Anaphylaxis	< 1 hour	+	

7	73	Female	Other drugs allergy	Systemic (cardiorespiratory, but no skin reaction)	Unknown	–	–


*CSU: Chronic spontaneous urticarial, LA: Local anesthetic

